# Renal function and lipid metabolism are major predictors of circumpapillary retinal nerve fiber layer thickness—the LIFE-Adult Study

**DOI:** 10.1186/s12916-021-02064-8

**Published:** 2021-09-07

**Authors:** Franziska G. Rauscher, Mengyu Wang, Mike Francke, Kerstin Wirkner, Anke Tönjes, Christoph Engel, Joachim Thiery, Peter Stenvinkel, Michael Stumvoll, Markus Loeffler, Tobias Elze, Thomas Ebert

**Affiliations:** 1 Leipzig Research Centre for Civilization Diseases (LIFE), Leipzig University, Leipzig, Germany; 2 Institute for Medical Informatics, Statistics, and Epidemiology (IMISE), Leipzig University, Leipzig, Germany; 3 Schepens Eye Research Institute, Harvard Medical School, Boston, MA USA; 4 Medical Department III – Endocrinology, Nephrology, Rheumatology, University of Leipzig Medical Center, Leipzig, Germany; 5 Institute of Laboratory Medicine, Clinical Chemistry and Molecular Diagnostics, Leipzig University, Leipzig, Germany; 6 Department of Clinical Science, Intervention and Technology, Division of Renal Medicine, Karolinska Institutet, Stockholm, Sweden

**Keywords:** Retinal nerve fiber layer, Glaucoma, Biomarkers, Renal function, Optical coherence tomography, Lipid profile, LDL cholesterol, HDL cholesterol, eGFR, Cystatin C, Apolipoprotein B, Apolipoprotein A1

## Abstract

**Background:**

Circumpapillary retinal nerve fiber layer thickness (cpRNFLT) as assessed by spectral domain optical coherence tomography (SD-OCT) is a new technique used for the detection and evaluation of glaucoma and other optic neuropathies. Before translating cpRNFLT into clinics, it is crucially important to investigate anthropometric, biochemical, and clinical parameters potentially affecting cpRNFLT in a large population-based dataset.

**Methods:**

The population-based LIFE-Adult Study randomly selected 10,000 participants from the population registry of Leipzig, Germany. All participants underwent standardized systemic assessment of various cardiometabolic risk markers and ocular imaging, including cpRNFLT measurement using SD-OCT (Spectralis, Heidelberg Engineering). After employing strict SD-OCT quality criteria, 8952 individuals were analyzed. Multivariable linear regression analyses were used to evaluate the independent associations of various cardiometabolic risk markers with sector-specific cpRNFLT. For significant markers, the relative strength of the observed associations was compared to each other to identify the most relevant factors influencing cpRNFLT. In all analyses, the false discovery rate method for multiple comparisons was applied.

**Results:**

In the entire cohort, female subjects had significantly thicker global and also sectoral cpRNFLT compared to male subjects (*p* < 0.05). Multivariable linear regression analyses revealed a significant and independent association between global and sectoral cpRNFLT with biomarkers of renal function and lipid profile. Thus, thinner cpRNFLT was associated with worse renal function as assessed by cystatin C and estimated glomerular filtration rate. Furthermore, an adverse lipid profile (i.e., low high-density lipoprotein (HDL) cholesterol, as well as high total, high non-HDL, high low-density lipoprotein cholesterol, and high apolipoprotein B) was independently and statistically significantly related to thicker cpRNFLT. In contrast, we do not observe a significant association between cpRNFLT and markers of inflammation, glucose homeostasis, liver function, blood pressure, or obesity in our sector-specific analysis and globally.

**Conclusions:**

Markers of renal function and lipid metabolism are predictors of sectoral cpRNFLT in a large and deeply phenotyped population-based study independently of previously established covariates. Future studies on cpRNFLT should include these biomarkers and need to investigate whether incorporation will improve the diagnosis of early eye diseases based on cpRNFLT.

**Supplementary Information:**

The online version contains supplementary material available at 10.1186/s12916-021-02064-8.

## Background

Retinal nerve fiber layer (RNFL) defects are early signs of glaucoma and optic disc deformation [1]. RNFL thickness is, therefore, a major tool in the evaluation of glaucoma and other optic neuropathies [2]. Spectral domain optical coherence tomography (SD-OCT) is an appropriate [3], non-invasive, in vivo technique for the analysis of the optic nerve, and recent advances have allowed improved image quality for circumpapillary RNFL thickness (cpRNFLT) [4]. Very recently, cpRNFLT has been associated with distinct, basic anthropometric, and biochemical measures in different studies. For instance, Ho et al. [5] demonstrate a positive association of global cpRNFLT with low-density lipoprotein (LDL) cholesterol and a negative correlation with diabetes prevalence in three different Asian ethnic cohorts. Furthermore, age and a history of stroke or hypertension were negatively, whereas smoking status was positively, related to global cpRNFLT in a cross-sectional meta-analysis of eight European, population-based studies [6]. In contrast, Lamparter et al. [7] did not find an independent association between global cpRNFLT and cardiovascular disease in multivariable analyses in the Gutenberg Health Study. Taken together, the association of global cpRNFLT shows conflicting results with the presence of cardiometabolic disease states. Before translating the cpRNFLT method into clinics, it is important to investigate anthropometric, biochemical, and clinical parameters potentially affecting cpRNFLT independent of other well-established predictors, i.e., age, sex, and scan radius. Furthermore, other factors influencing cpRNFLT need to be carefully defined to aid the early diagnosis of eye diseases and to prevent misclassification of impaired cpRNFLT due to other clinical and biochemical biomarkers. However, previous studies on cpRNFLT show the following limitations: they (a) included cohorts of smaller sample size; (b) have analyzed global cpRNFLT but not sector-specific data; (c) excluded subjects with different cardiometabolic disease states, e.g., type 2 diabetes or hypertension; (d) did not include a wide range of anthropometric, biochemical, and cardiometabolic markers and other patient-level data; and (e) did not use thoroughly adjusted multivariable models to investigate the independent predictors of cpRNFLT.

We, therefore, investigated a large panel of different anthropometric and cardiometabolic biomarkers and a wide range of clinical phenotypes and their associations with the sector-specific cpRNFLT profile measured by SD-OCT in a large (*N* = 8952 subjects), unselected, and deeply phenotyped population-based study in Germany. We have applied a highly standardized ophthalmologic and non-ophthalmologic investigation procedure and statistical adjustment with correction for multiple testing.

## Methods

### Participants

This analysis is part of the population-based LIFE-Adult Study conducted by the Leipzig Research Centre for Civilization Diseases at Leipzig University between August 2011 and November 2014 [8]. The LIFE-Adult Study includes 10,000 randomly selected participants from the population registry of just over half a million inhabitants of Leipzig, a city located in the east of Germany.

The LIFE-Adult Study recruitment was performed in an age- and sex-stratified manner mainly focusing on subjects with an age between 40 and 79 years [8]. For this purpose, the overall population consisted of 9600 subjects between 40 and 79 years of age, as well as 400 subjects between 19 and 39 years of age. Each age interval (by decade) was balanced with respect to the number of subjects and sex. The study was approved by the Ethical Committee at the Medical Faculty of Leipzig University (approval number: 263-2009-14122009) and adheres to the Declaration of Helsinki and all federal and state laws. Prior to inclusion, informed written consent was obtained from all participants.

### Data collection/inclusion and exclusion criteria

During the baseline examination, study participants were deeply phenotyped, including ophthalmological image data, structured interviews, questionnaires, physical examinations, and blood and urine tests [8]. As part of the ophthalmic assessment, SD-OCT imaging (Spectralis, Heidelberg Engineering, Heidelberg, Germany) was performed, yielding cpRNFLT scans around the optic nerve head. The location of the cpRNFLT circle and the coordinate system have been described previously [4]. We excluded subjects with missing SD-OCT scans (excluded *N* = 931) or SD-OCT scans using the following quality criteria: (1) B-scan number per location < 50, (2) signal to noise ratio < 20 dB, and (3) missing or unreliable RNFLT A-scans > 5% (excluded *N* = 117). For the remaining 8952 subjects, one eye was randomly selected if both eyes of an included subject were reliable [4]. For validation analyses, we classified optic nerve head (ONH) abnormalities if any of the following were present: excavation (suspected glaucoma [i.e., violation of the inferior-superior-nasal-temporal rule, vertically oval with cup-to-disc ratio > 0.7], optic disc pit, or coloboma of the optic disc), optic disc hemorrhage, neovascularization, optic atrophy, sectoral paleness, ONH swelling, papilledema, or optic disc drusen [4]. Furthermore, patient information on a previous diagnosis of glaucoma, as well as glaucoma medication, were collected.

### Anthropometric and biochemical markers

Classical anthropometric (e.g., body mass index [BMI], waist and hip circumferences, blood pressure) measurements were assessed according to standardized procedures by trained study nurses. In all subjects, fasting blood samples were drawn routinely and a panel of laboratory tests was performed on the day of sample collection [8]. The biomarkers of the panel have been described previously [8] and included measurements of total cholesterol, high-density lipoprotein (HDL), low-density lipoprotein (LDL) cholesterol, triglycerides (TG), apolipoprotein (apo) B, apoA1, lipoprotein (Lp) (a), glucose, insulin, glycated hemoglobin (HbA1c), liver enzymes, interleukin-6, high-sensitivity C-reactive protein (hsCRP), cystatin C, and urinary albumin and creatinine, all being quantified in a central lab by standard methods [8]. In all subjects, the estimated glomerular filtration rate (eGFR) was calculated using the cystatin C-based chronic kidney disease (CKD) epidemiology collaboration equation [9]. As the aim of the current study was to investigate the associations between several cardiometabolic risk markers and cpRNFLT, we have used only the cystatin C-based equation which has been recently reported as the best equation for the assessment of cardiovascular risk [10]. CKD status was defined as a urinary albumin/creatinine ratio ≥ 30 mg/g and/or a decreased eGFR < 60 ml/min/1.73 m^2^, and the cohort was divided into five eGFR categories (i.e., G1-G5 combining G3a and G3b into one G3 category), as well as four CKD risk categories (i.e., low, moderately increased, high, and very high risk), according to KDIGO [11].

### Statistical analysis

All statistical analyses were performed in R environment using version 3.5 (R Foundation for Statistical Computing, Vienna, Austria). For comparisons between female and male subjects, the unpaired Student *t*-test (for continuous variables) or chi-squared test (for categorical variables) were used, respectively.

As a next step, we examined the associations of various anthropometric, as well as cardiometabolic, biomarkers on the sectoral RNFLT. For this purpose, multivariable linear regression analyses were carried out for individual markers adjusted for age, sex, and scanning circle radius in all models. Using these covariates as independent variables in the respective models, the association of each marker with sectoral cpRNFLT (dependent variable) were separately calculated for the temporal (T), supero-temporal (TS), supero-nasal (NS), nasal (N), infero-nasal (NI), and infero-temporal (TI) sectors, and globally (G).

Scanning circle radius was included as an independent variable in all models, since eye size and optical characteristics of the human lens confound the cpRNFLT measurement [12–14]. The true scanning circle radius (mm) is estimated from the focus settings used by the Spectralis machine, according to a widely used model [15]. As sex [4] and age [14] were shown to affect cpRNFLT, these markers were also included as independent covariates in each model.

We next sought to compare the relative strength of the associations of all biomarkers with sectoral cpRNFLT. Therefore, a sectoral and global heatmap of standardized *β* values from multivariable analyses for each sectoral cpRNFLT was produced for all biomarkers, and standardized *β* values were employed in the figure’s color code representing the strength of each association.

As a sensitivity analysis, we further validated the results of the linear regression analyses for lipid markers with sectoral cpRNFLT by stratifying the cohort into subjects on statin treatment compared to non-statin users. For this purpose, we used the Anatomical Therapeutic Chemical (ATC) classification codes to extract participants treated with 3-hydroxy-3-methylglutaryl coenzyme A reductase inhibitors (i.e., statins), thereby reducing cholesterol synthesis. To investigate the potential mediating effects of smoking status on the association between cpRNFLT and the lipid profile, the Bayesian information criterion difference (ΔBIC) was computed for model comparisons. For this purpose, two different linear regression models were calculated with age, sex, measurement radius, and the respective lipid marker, as regressors (model A), as well as an additional model comprising of model A + smoking status (model B). The BIC difference (ΔBIC) was calculated by ΔBIC = BIC_model A_ − BIC_model B_. A ΔBIC > 2 was regarded as statistically relevant according to Madrigal-González et al. [16], as well as Kass and Raftery [17].

In all other analyses, a *p* value < 0.05 was considered as statistically significant. The false discovery rate (FDR) method was applied to correct all *p* values for multiple comparisons.

## Results

### Baseline characteristics of the entire study population (*N* = 8952)

Baseline characteristics of cohort stratified by sex are shown in Table 1. The mean ± standard deviation age of the total population was 57.8 ± 12.4 years. In the entire cohort, about 1243 (13.9%) patients had a diabetes. Hypertension was present in 4444 (49.6%) subjects, and 1130 (12.6 %) participants received statin treatment. Moreover, 1875 (20.9%) subjects were current smokers. Female subjects had lower markers of obesity (i.e., BMI, waist-to-hip ratio), glucose homeostasis (i.e., fasting glucose, fasting insulin, HbA1c), blood pressure, albuminuria, interleukin﻿-6, and liver enzymes compared to male participants (all *p* < 0.05; Table 1). In contrast, women had higher total cholesterol, HDL cholesterol, apoA1, Lp(a), and hsCRP, compared to men (all *p* < 0.05; Table 1). Renal function (eGFR), LDL cholesterol, and alkaline phosphatase did not depend on sex (all *p* > 0.05; Table 1). The mean thicknesses for the respective sectoral cpRNFLT and global cpRNFLT are depicted in Table 2. Female subjects had significantly thicker global cpRNFLT and also regional cpRNFLT in all sectors compared to male participants (all *p* < 0.05; Table 2), except NS sector (*p* = 0.067; Table 2).

**Table 1:** Baseline characteristics of the entire study population stratified by sex (*N* = 8952)

	Female subjects	Male subjects
Total, *N*	4665	4287
Age (years)	57.0 ± 12.2	57.9 ± 12.6*
Diabetes, *N* (%)	521 (11.2)	722 (16.8)*
Smoker, *N* (%)	910 (19.5)	965 (22.5)*
Hypertension, *N* (%)	2094 (44.9)	2350 (54.8)*
Statin therapy, *N* (%)	423 (9.1)	707 (16.5)*
CKD risk groups, *N* (%)		
Low CKD risk	3911 (83.8)	3526 (82.2)*
Moderate CKD risk	518 (11.1)	506 (11.8)
High CKD risk	117 (2.5)	144 (3.4)*
Very high CKD risk	42 (0.9)	53 (1.2)
BMI (kg/m^2^)	27.1 ± 5.4	27.6 ± 4.2*
WHR	0.9 ± 0.1	1.0 ± 0.1*
SBP (mmHg)	125.1 ± 17.2	131.7 ± 15.5*
DBP (mmHg)	73.9 ± 9.6	76.7 ± 9.9*
Cystatin C (mg/l)	0.9 ± 0.2	1.0 ± 0.2*
eGFR_Cys_ (ml/min per 1.73m^2^)	86.0 ± 19.1	86.8 ± 20.2
Urinary albumin-creatinine ratio (mg/g)	18.6 ± 118.6	29.6 ± 214.4*
Fasting glucose (mmol/l)	5.5 ± 1.1	5.9 ± 1.2*
Fasting insulin (pmol/l)	62.6 ± 113.7	70.6 ± 54.6*
HbA1c (%)	5.4 ± 0.5	5.4 ± 0.6*
Total cholesterol (mmol/l)	5.7 ± 1.1	5.4 ± 1.1*
HDL cholesterol (mmol/l)	1.8 ± 0.5	1.4 ± 0.4*
Non-HDL cholesterol (mmol/l)	3.9 ± 1.1	4.0 ± 1.1*
LDL cholesterol (mmol/l)	3.5 ± 1.0	3.5 ± 1.0
TG (mmol/l)	1.2 ± 0.7	1.6 ± 1.3*
ApoA1 (g/l)	1.8 ± 0.3	1.5 ± 0.2*
ApoB (g/l)	1.1 ± 0.3	1.1 ± 0.3*
Lp(a) (g/l)	0.2 ± 0.3	0.2 ± 0.3*
hsCRP (mg/l)	3.1 ± 6.1	2.5 ± 4.7*
IL-6 (ng/l)	3.5 ± 4.7	3.8 ± 4.9*
ALAT (μkat/l)	0.4 ± 0.2	0.5 ± 0.3*
ASAT (μkat/l)	0.4 ± 0.1	0.5 ± 0.2*
AP (μkat/l)	1.2 ± 0.4	1.1 ± 0.3
GGT (μkat/l)	0.5 ± 0.6	0.8 ± 0.9*

Values for mean ± standard deviation or total number (percentage) are shown. *p* values were assessed by the *t* test or chi-squared test and corrected for multiple testing based on the false discovery rate method. *p < 0.05 for female vs. male subjects

*ALAT*, alanine aminotransferase; *AP*, alkaline phosphatase; *ApoA1*, apolipoprotein A1; *ApoB*, apolipoprotein B; *ASAT*, aspartate aminotransferase; *BMI*, body mass index; *DBP*, diastolic blood pressure; *eGFR*_*Cys*_, cystatin C-based estimated glomerular filtration rate; *GGT*, gamma-glutamyltransferase; *HbA1c*, glycated hemoglobin A1c; *HDL*, high-density lipoprotein; *hsCRP*, high-sensitivity C-reactive protein; *IL*, interleukin; *LDL*, low-density lipoprotein; *Lp(a)*, lipoprotein(a); *SBP*, systolic blood pressure; *TG*, triglycerides; *WHR*, waist-to-hip ratio

**Table 2:** RNFL thickness of the entire study population stratified by sex (*N* = 8952)

	Female subjects	Male subjects
Total, *N*	4665	4287
G (μm)	95.3 ± 11.0	93.6 ± 11.6*
T (μm)	72.0 ± 13.1	69.2 ± 12.5*
TS (μm)	130.4 ± 20.6	129.4 ± 21.2*
TI (μm)	141.0 ± 21.2	136.8 ± 22.1*
N (μm)	70.8 ± 15.7	69.8 ± 15.6*
NS (μm)	102.3 ± 22.2	103.2 ± 22.8*
NI (μm)	102.7 ± 22.9	101.2 ± 23.1*

The total number of included subjects, as well as the mean ± standard deviation of averaged retinal nerve fiber layer (RNFL) thickness over each of the six regions, is depicted

*p* values were assessed by the *t* test and corrected for multiple testing based on the false discovery rate method. *p < 0.05 for female vs. male subjects

*T*, temporal; *TS*, supero-temporal; *NS*, supero-nasal; *N*, nasal; *NI*, infero-nasal; *TI*, infero-temporal

### Associations between sector-specific cpRNFLT and cardiometabolic biomarkers

**Fig. 1:**
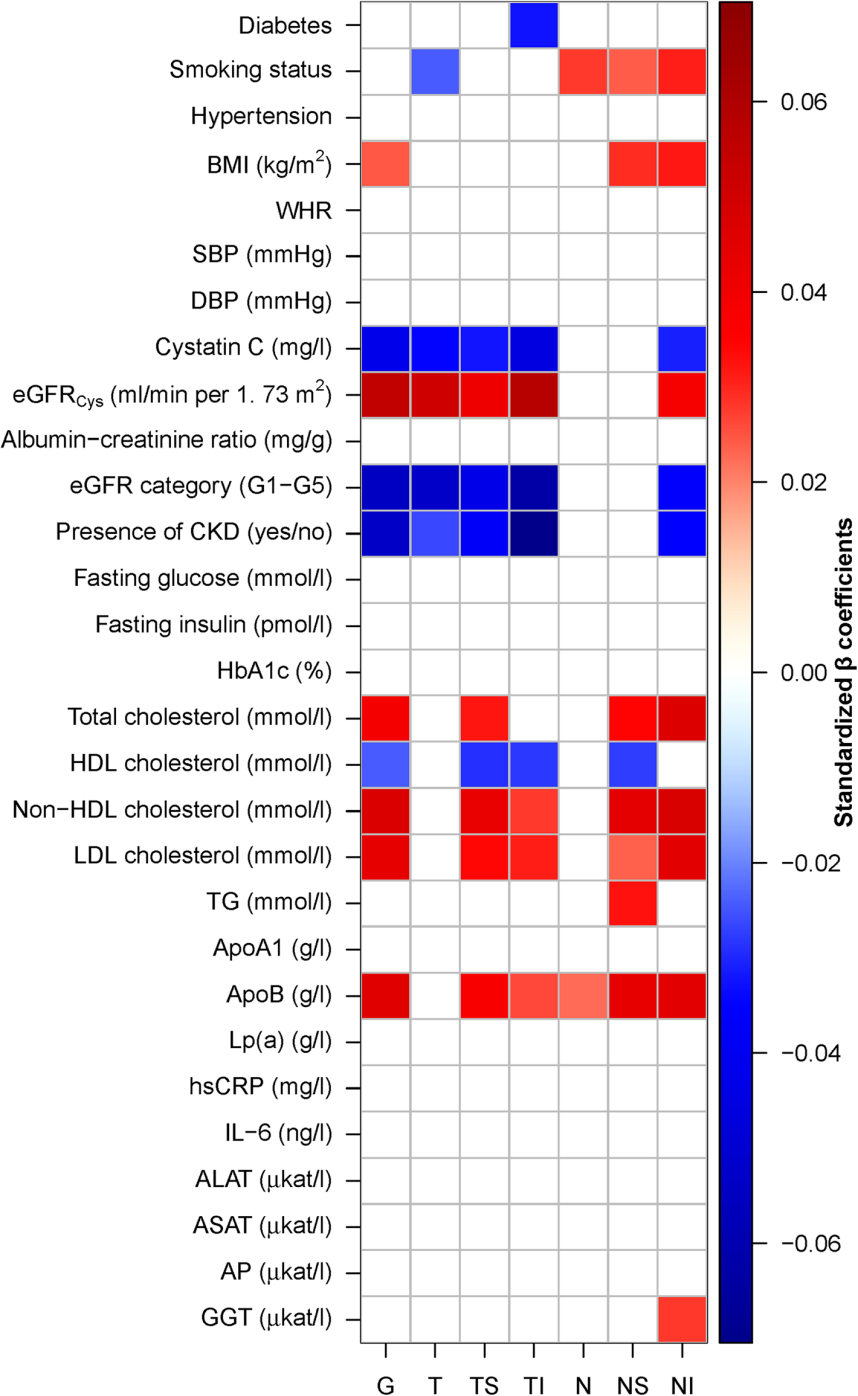
Heatmap of standardized *β* coefficients for all investigated biomarkers and global, as well as sectoral, circumpapillary retinal nerve fiber layer thickness (cpRNFLT). Separate multivariable linear regression analyses were carried out for each of the biomarkers (independent variable) and the respective sectoral or global cpRNFLT (dependent variable). All multivariable models were adjusted for age, sex, and scanning circle radius. The false-positive discovery rate method was applied to correct *p* values for multiple comparisons. If the linear regression models did not show an overall significance (indicating that the standardized *β* is not valid in this sector), a white (empty) square is depicted. For all significant sectors, strength as assessed by standardized *β*, as well as the direction, of the associations are color-coded. Thus, positive (in red/warmer colors) and negative (in blue/cooler colors) associations are shaded based on the respective standardized *β* coefficients. Abbreviations: ALAT, alanine aminotransferase; AP, alkaline phosphatase; ApoA1, apolipoprotein A1; ApoB, apolipoprotein B; ASAT, aspartate aminotransferase; BMI, body mass index; CKD, chronic kidney disease; DBP, diastolic blood pressure; eGFR_Cys_, cystatin C-based estimated glomerular filtration rate; GGT, gamma-glutamyltransferase; HbA1c, glycated hemoglobin A1c; HDL, high-density lipoprotein; hsCRP, high-sensitivity C-reactive protein; IL, interleukin; LDL, low-density lipoprotein; Lp(a), lipoprotein(a); SBP, systolic blood pressure; TG, triglycerides; WHR, waist-to-hip ratio. Optic nerve head sectors: N, nasal sector; NI, infero-nasal sector; NS, supero-nasal sector; T, temporal sector; TI, infero-temporal sector; TS, supero-temporal sector; G, global (mean overall)

Visual inspection of selected clinical parameters of renal function (i.e., eGFR) and lipid profile (i.e., HDL cholesterol, non-HDL cholesterol) revealed that there is a linear relationship between these biomarkers and global cpRNFLT (Additional file 1: Fig. S1). Therefore, linear regression models were used in all subsequent analyses. Multivariable associations with cpRNFLT averaged across each of the six respective sectors, as well as the global mean, were separately calculated for each biomarker and sector with adjustment for potential covariates found in previous analyses, i.e., age [6, 14], sex [4], and scanning circle radius [12–14]. A significant and independent association between global cpRNFLT and BMI, cystatin C, eGFR, eGFR category, and presence of CKD, as well as total cholesterol, HDL cholesterol, non-HDL cholesterol, LDL cholesterol, and ApoB, was found, respectively (Table 3, Fig. 1). These results indicate that renal function and lipid profile contribute to cpRNFLT. As correction for multiple comparisons potentially increases type II errors, analysis was repeated without FDR correction and revealed comparable results (data not shown), which demonstrates that the presence and absence of effects in our results are distinct enough from each other that shifting significance criteria within the limits of multiple comparison adjustments does not change any conclusions. When the entire analysis was further adjusted for SD-OCT-derived clinical and subclinical ONH abnormalities (including glaucoma and other ONH diseases), as well as patient-reported glaucoma diagnosis and medication, the associations of all investigated biomarkers and cpRNFLT were virtually unchanged in terms of effect size and direction (Additional file 2: Table S1).

**Table 3:** Sectoral multivariable linear regression analyses for cardiometabolic biomarkers and cpRNFLT in all subjects (*N* = 8952)

Sectors	Global		T		TS		TI		N		NS		NI	
	***B***	***p***_**adjusted**_	***B***	p_**adjusted**_	***B***	p_**adjusted**_	***B***	***p***_**adjusted**_	***B***	***p***_**adjusted**_	***B***	***p***_**adjusted**_	***B***	***p***_**adjusted**_
Diabetes	− 0.81	0.056	− 0.79	0.114	− 1.18	0.115	− 2.05	**0.013**	− 0.57	0.332	− 0.50	0.552	− 0.05	0.940
Smoking status	0.58	0.053	− 0.75	**0.047**	0.41	0.450	0.63	0.295	1.05	**0.033**	1.31	**0.047**	1.70	**0.024**
Hypertension	− 0.41	0.235	− 0.26	0.671	− 0.02	0.965	− 0.85	0.235	− 0.83	0.140	− 0.17	0.911	− 0.14	0.911
BMI (kg/m^2^)	0.06	**0.035**	− 0.03	0.294	0.08	0.152	0.07	0.201	0.04	0.254	0.14	**0.018**	0.15	**0.014**
WHR	2.08	0.330	− 3.62	0.212	1.13	0.780	0.96	0.780	3.63	0.258	8.40	0.144	6.12	0.212
SBP (mmHg)	− 0.01	0.232	− 0.02	0.220	− 0.02	0.220	− 0.02	0.232	− 0.01	0.282	0.00	0.820	0.02	0.220
DBP (mmHg)	0.00	0.927	− 0.02	0.605	0.01	0.927	0.00	0.927	0.00	0.927	− 0.03	0.605	0.05	0.312
Cystatin C (mg/l)	− 2.18	**< 0.001**	− 2.04	**0.006**	− 3.06	**0.008**	− 4.47	**< 0.001**	− 0.75	0.358	− 1.24	0.337	− 3.23	**0.008**
eGFR_Cys_ (ml/min per 1.73 m^2^)	0.03	**< 0.001**	0.03	**0.001**	0.04	**0.006**	0.06	**< 0.001**	0.01	0.238	0.01	0.590	0.04	**0.007**
Albumin-creatinine ratio (mg/g)	Cave:	0.916	0.00	0.916	0.00	0.916	0.00	0.916	0.00	0.686	0.00	0.953	0.00	0.916
eGFR category (G1–G5)	− 0.93	**< 0.001**	− 1.04	**< 0.001**	− 1.36	**0.001**	− 2.07	**< 0.001**	− 0.18	0.550	− 0.36	0.467	− 1.27	**0.004**
Presence of CKD (yes/no)	− 2.06	**< 0.001**	− 1.16	**0.026**	− 2.70	**0.001**	− 5.24	**< 0.001**	− 0.88	0.133	− 1.65	0.059	− 2.80	**0.002**
Fasting glucose (mmol/l)	0.04	0.731	− 0.14	0.453	− 0.11	0.661	− 0.23	0.453	0.19	0.453	0.28	0.453	0.21	0.453
Fasting insulin (pmol/l)	0.00	0.409	0.00	0.458	0.00	0.260	0.00	0.437	0.00	0.868	0.01	0.223	0.00	0.868
HbA1c (%)	0.19	0.612	0.29	0.612	− 0.05	0.905	− 0.08	0.905	0.43	0.612	− 0.33	0.612	0.43	0.612
Total cholesterol (mmol/l)	0.42	**< 0.001**	0.03	0.803	0.63	**0.004**	0.36	0.102	0.28	0.096	0.74	**0.002**	1.00	**< 0.001**
HDL cholesterol (mmol/l)	− 0.58	**0.044**	− 0.14	0.760	− 1.29	**0.034**	− 1.30	**0.034**	− 0.01	0.979	− 1.32	**0.034**	− 0.41	0.617
Non-HDL cholesterol (mmol/l)	0.49	**< 0.001**	0.06	0.623	0.82	**< 0.001**	0.56	**0.009**	0.27	0.086	0.91	**< 0.001**	1.01	**< 0.001**
LDL cholesterol (mmol/l)	0.51	**< 0.001**	0.24	0.107	0.74	**0.002**	0.71	**0.004**	0.26	0.121	0.56	**0.032**	1.08	**< 0.001**
TG (mmol/l)	0.22	0.082	− 0.16	0.289	0.44	0.075	0.10	0.635	0.16	0.370	0.70	**0.013**	0.51	0.075
ApoA1 (g/l)	− 0.66	0.190	− 1.04	0.126	− 1.46	0.149	− 1.81	0.126	− 0.17	0.907	− 0.03	0.976	0.43	0.856
ApoB (g/l)	1.92	**< 0.001**	0.13	0.798	2.92	**0.001**	2.14	**0.016**	1.32	**0.037**	3.65	**< 0.001**	3.84	**< 0.001**
Lp(a) (g/l)	0.21	0.887	0.22	0.887	− 0.21	0.887	0.39	0.887	0.07	0.887	0.58	0.887	0.20	0.887
hsCRP (mg/l)	0.03	0.477	0.00	0.919	0.04	0.477	− 0.01	0.919	0.02	0.612	0.06	0.477	0.06	0.477
IL-6 (ng/l)	0.00	0.998	− 0.01	0.920	0.04	0.920	− 0.03	0.920	− 0.03	0.920	0.07	0.920	0.00	0.998
ALAT (μkat/l)	0.18	0.982	− 0.44	0.982	− 0.03	0.982	0.51	0.982	0.23	0.982	− 0.02	0.982	1.37	0.961
ASAT (μkat/l)	− 0.47	0.709	− 0.86	0.709	− 0.14	0.920	− 1.03	0.709	− 0.74	0.709	0.60	0.853	0.12	0.920
AP (μkat/l)	− 0.13	0.894	− 0.30	0.894	− 0.38	0.894	0.09	0.894	− 0.22	0.894	0.09	0.894	0.16	0.894
GGT (μkat/l)	0.01	0.918	− 0.30	0.287	− 0.16	0.773	0.06	0.918	− 0.13	0.773	0.33	0.597	0.80	**0.045**

For each of the six cpRNFL sectors, a linear regression model was calculated with age, sex, and measurement radius, as well as the respective biomarker, as regressors. Unstandardized *B* coefficients, i.e., slope, and corresponding *p* values (corrected for multiple testing based on the false discovery rate method) for the respective cardiometabolic biomarkers are depicted. Abbreviations are indicated in Tables 1 and 2. *p* values marked in bold indicate significant association in multivariate analysis

#### Renal function and sector-specific cpRNFLT

The associations of cpRNFLT with markers of renal function remained virtually the same in all three temporal sectors (i.e., T, TS, TI), as well as NI (Table 3, Fig. 1). In more detail, global, temporal, and NI cpRNFLT were significantly and independently associated with cystatin C, eGFR, eGFR category, and CKD status (Fig. 1). Thus, eGFR was positively associated with cpRNFLT in the respective sectors indicating that better renal function is linked to thicker cpRNFLT (Fig. 1). Conversely, cystatin C, eGFR category, and CKD status were inversely related to cpRNFLT in the respective sectors with the strongest associations observed in TI and globally (Fig. 1). Per 1 mg/l increase in cystatin C, there was a decrease of − 2.2 μm in global cpRNFLT (Table 3).

#### Lipid profile and sector-specific cpRNFLT

Lipid markers were statistically significantly and independently associated with sector-specific cpRNFLT for total cholesterol, HDL cholesterol, non-HDL cholesterol, LDL cholesterol, and ApoB, in sectors TS, TI, NS, NI, and globally (Table 3, Fig. 1).

For ApoB-containing lipid particles (i.e., non-HDL cholesterol, LDL cholesterol, total cholesterol), a positive correlation between each lipid measure and sector-specific cpRNFLT was found except for T and N, and the strongest associations were observed for non-HDL cholesterol and LDL cholesterol (Fig. 1). In contrast, HDL cholesterol was negatively and statistically significantly related to cpRNFLT in TS, NS, TI, and globally (Fig. 1) indicating that HDL cholesterol is inversely related to cpRNFLT compared to ApoB-containing lipid particles. Thus, an adverse lipid profile (i.e., high total cholesterol, high non-HDL cholesterol, high LDL cholesterol, high ApoB, low HDL cholesterol) is independently and statistically significantly related to thicker cpRNFL (Fig. 1). Clinically, per 1 mmol/l increase in non-HDL cholesterol, there was an increase of 0.5 μm in global cpRNFLT (Table 3).

#### Other biomarkers and sector-specific cpRNFLT

Current smoking was significantly and independently related to cpRNFLT in sector T and all nasal sectors (i.e., NS, NI, N) (Table 3, Fig. 1). The positive association of global cpRNFLT and BMI was also confirmed in sectors NS and NI (Table 3, Fig. 1). Furthermore, diabetes status and gamma-glutamyltransferase were significantly associated with TI and NI of the cpRNFLT, respectively (Table 3, Fig. 1).

Although we have not found an association between cpRNFLT and inflammatory markers (Table 3, Fig. 1), inflammation might mediate the observed results. Therefore, all analyses were additionally adjusted for hsCRP, and results remained virtually unchanged with additional adjustment for inflammation (Additional file 3: Table S2). Furthermore, when using interleukin-6 instead of hsCRP, comparable associations were observed (data not shown).

### Sensitivity analyses—CKD risk groups, statin treatment, smoking status, and glaucoma status

To verify our results obtained from multivariable analyses for markers of renal function, we have stratified the entire cohort based on the CKD risk groups according to KDIGO [11]. Subjects with advanced CKD (i.e., moderate/high/very high risk) had a significantly thinner global and sectoral cpRNFL compared to participants without CKD (low risk; Additional file 4: Table S3) supporting our findings from regression models (Table 3).

Using a similar approach, patients on statin treatment showed a significantly thinner global, as well as temporal, cpRNFL compared to non-statin users (Additional file 5: Table S4). When multivariable analyses of cpRNFLT and lipid profile were stratified by statin usage, associations between lipid profile and cpRNFLT remained similar for non-statin users compared to the entire cohort (Additional file 6: Table S5). In contrast, patients on statin treatment only showed a positive, significant, and independent association of non-HDL cholesterol with cpRNFLT in sector NS, as well as globally (Additional file 6: Table S5).

We further investigated whether smoking status mediates our observed results of cpRNFLT with lipid profile as both smoking and lipid profile were counterintuitively positively associated with cpRNFLT. Using Bayesian information criterion difference (ΔBIC), smoking status does not have an additive effect on linear regression models for cpRNFLT with adverse lipid markers (all ΔBIC < 2; Additional file 7: Table S6).

If including several possible confounders of cpRNFLT (i.e., age, sex, scan radius, smoking status, eGFR, non-HDL cholesterol, and hsCRP) in one regression model for each sector and globally, age, sex, scan radius, eGFR, and non-HDL cholesterol were found to be the most important factors independently contributing to cpRNFLT changes (data now shown). Collectively, these data support our findings of independent associations of renal function, as well as lipid profile, with cpRNFLT (Table 3).

As cpRNFL thinning is associated with glaucoma [18], we next sought to investigate whether an adverse lipid profile is similarly associated with cpRNFLT in patients with ONH abnormalities, self-reported glaucoma diagnosis, and/or glaucoma medication (*N* = 1180). In this subset, the associations of lipid markers and cpRNFLT were virtually unchanged (Additional file 8: Table S7) compared to the entire cohort (Table 3).

## Discussion

In the current study, we identify potential anthropometric and cardiometabolic markers which are independently and significantly associated with sector-specific cpRNFLT using a large dataset comprising of 10,000 deeply phenotyped subjects [8]. As we find that different renal and metabolic biomarkers are associated with cpRNFLT, translational implementation of cpRNFLT profiles to clinics and research requires adjustment for these confounders in future studies.

We report that a cystatin C-based calculation of eGFR, i.e., currently the best equation for assessment of cardiovascular risk [10], was independently associated with cpRNFLT. To the best of our knowledge, no previous large population-based study has investigated the effect of renal function on sectoral cpRNFLT. A small study of 60 patients with diabetic retinopathy vs. 20 healthy controls also showed that thinner RNFLT is correlated with the increase in serum urea and creatinine, i.e., two other surrogate markers of renal dysfunction [19]. Furthermore, data from a Chinese cohort of 1408 patients with type 2 diabetes also revealed a positive relation between eGFR and cpRNFLT [20]. Despite some similar correlations [21], we refrain from comparing our SD-OCT-derived data to some previous studies evaluating cpRNFLT by fundus photography. In summary, thinner cpRNFLT is a feature of CKD, an established risk factor for the progression to end-stage kidney disease (ESKD) and mortality [10, 22]. As our data show an independent association between renal function and cpRNFLT, future studies need to investigate whether cpRNFLT could predict the risk of progressive CKD and/or mortality, as well as mechanistically identify whether impaired renal function impairs RNFL characteristics similar to brain lesions, e.g., white matter lesions, silent cerebral infarction, microbleeds, and brain atrophy [23].

A distinct set of adverse lipid markers was significantly and positively associated with cpRNFLT in our cohort. Thus, total cholesterol, non-HDL cholesterol, LDL cholesterol, and circulating apoB are positive predictors of sectoral cpRNFLT in most sectors except for T. It is interesting to note that apoB is the primary apolipoprotein of chylomicrons, very low-density lipoproteins, intermediate-density lipoproteins, and LDL particles, collectively summarized as non-HDL cholesterol. Thus, similar results in our models for non-HDL cholesterol and apoB validate our findings. Furthermore, LDL cholesterol is the main component of total cholesterol, and therefore, the association of total cholesterol with cpRNFLT is weaker but still comparable to apoB, non-HDL cholesterol, and LDL cholesterol. The aforementioned positive associations of adverse lipid particles with cpRNFLT in our large cohort are of interest, as these lipid particles, for instance, total cholesterol [24] and LDL cholesterol [25], also predict increased vascular and overall mortality. Furthermore, HDL cholesterol is associated with a beneficial cardiovascular risk profile and reduced mortality [26]. In accordance with our data on the positive correlation of cpRNFLT and adverse apoB-containing lipid particles, HDL cholesterol is negatively associated with sectoral cpRNFLT. Collectively, our data indicate that an adverse lipid profile as assessed by high apoB, high non-HDL cholesterol, high LDL cholesterol, and low HDL cholesterol is independently and statistically significantly related to a thicker cpRNFLT. Importantly, the presence of SD-OCT-derived clinical and subclinical ONH abnormalities (including glaucoma and other ONH diseases), as well as patient-reported glaucoma diagnosis and medication, did not influence the observed results (Additional file 8: Table S7). Pathophysiologically, the retina is capable of the rapid uptake of distinct cholesterol particles from the circulation [27]. Furthermore, many relevant proteins and receptors necessary for uptake, transport, metabolism, synthesis, and efflux of cholesterol and other lipid molecules [28] are expressed in cells of the human RNFL (i.e., ganglion cells and glia cell types such as astrocytes and Müller cells) [29, 30]. Moreover, lipid-lowering drugs, for instance, statins, are permeable to the blood-retinal barrier [31], and chronic (i.e., for 6 weeks) simvastatin treatment in mice decreases total retinal cholesterol content by 24% [31]. In more detail, simvastatin reduced retinal cholesterol biosynthesis and to a lesser extent increased retinal uptake of serum cholesterol leading to a reduced retinal cholesterol content [31]. Collectively, retinal lipid synthesis is likely to be the main driver of retinal cholesterol content, but circulating lipids and lipid-lowering compounds also significantly contribute to retinal cholesterol levels [31, 32]. Thus, increased circulating lipid levels hypothetically might induce lipid trafficking from the blood stream into the peripapillary retinal tissue and, concomitantly, increase the volume of cpRNFL by lipid accumulation. Additionally, dysregulated lipid particles could contribute to volume changes and accumulation of cholesterol deposits in the endothelial cells and pericytes of the vasculature around the optic nerve head. Furthermore, retention of lipoproteins in retinal layers potentially induces lipid modifications (i.e., oxidized lipoproteins, different forms of cholesterol) and adverse pro-inflammatory, pro-angiogenic downstream effects—a phenotype similar to atherosclerotic coronary artery disease [33]. It is important to note in this context that an adverse lipid profile cannot prevent glaucoma as both processes differ pathophysiologically. As a consequence, the decay of nerve fibers in glaucoma potentially could be clinically obscured in patients with an adverse lipid profile due to a net effect of “normal” cpRNFLT.

Our observed reduced lipid levels in the circulation, as well as decreased sectoral cpRNFLT, in statin-treated subjects compared to non-statin users (Additional file 5: Table S4), further support the hypothesis of a concentration-difference-driven accumulation of lipids in peripapillary retinal tissue and blood vessels.

Importantly, after stratifying the cohort into statin-treated subjects compared to non-statin users, the direction of the associations remained virtually the same (although statistical significance was partly lost in the statin-treated group) (Additional file 7: Table S6). Potential reasons for the observed effects include an altered lipid metabolism due to statin treatment; disassociation of the cpRNFLT connection to lipid profile by statin treatment, which cannot reverse the cpRNFLT phenotype (in contrast to the statin effects on lipid markers); and/or a reduced number (*N* = 1130) of included subjects in the subanalysis. As cpRNFL thinning is associated with neuropathies like glaucoma [34, 35] and systemic diseases like diabetes mellitus, even prior to the development of the typical retinal defects related to diabetic retinopathy [36–38], the lipid data in the entire cohort may seem counterintuitive. On the other hand, our lipid results are in analogy to the counterintuitive effect of smoking status on cpRNFLT. Here, smoking is also positively associated with the thickening of cpRNFLT in nasal sectors N, NS, and NI in our cohort, similar to an adverse lipid profile. Interestingly, these data are in accordance with the results from a recent meta-analysis [6] where a positive correlation between current or former smoking and global cpRNFLT was also found in the Rotterdam II and in the Montrachet Study [6]. A further small case-control study by Teberik [39] also shows numerically thicker RNFL in the three nasal sectors (but the statistical significance has been achieved only in the NI sector). As smoking potentially interferes with lipid profile [40], we investigated the mediating effects of smoking status and lipid profile. Here, smoking status did not interfere with the observed associations of cpRNFLT with the lipid profile (Additional file 7: Table S6) suggesting separate effects of both variables, i.e., smoking status and lipid profile, on cpRNFLT.

In contrast to the significant associations observed for renal function and dyslipidemia in relation to cpRNFLT, we did not observe significant correlations between cpRNFLT and markers of inflammation, glucose homeostasis, liver function, blood pressure, and obesity in our sector-specific analysis investigating six sectors and globally. While we are aware of the association of diabetes status with thinning of cpRNFLT in some [5, 41] but not all [7] studies, our large population study suggests that clinicians do not need to consider these cardiometabolic risk markers as potential confounders in sector-specific cpRNFLT examination.

Based on our results, it is tempting to speculate whether incorporating our novel identified RNFLT-linked biomarkers (i.e., renal function, lipid profile, smoking status) additional to conventional (i.e., age, sex, scanning circle radius) parameters will improve the diagnosis of early eye diseases based on cpRNFLT.

This study has several limitations: First, the study population predominantly consisted of European subjects, and therefore, the findings may not be generalizable to populations of different ethnicities. Secondly, the cross-sectional design of this study does not permit causal conclusions. However, strengths of the current study include a large number of deeply phenotyped subjects at a very high level of standardization, as well as a thorough statistical approach accounting for several important covariates.

## Conclusion

In conclusion, markers of renal function and lipid metabolism are independent predictors of sectoral cpRNFLT in a large and deeply phenotyped population-based study and should be included as important covariates in future studies on cpRNFLT.

## Supplementary Information

**Additional file 1: Figure S1.** Scatterplots illustrating the relationship between global circumpapillary retinal nerve fiber layer thickness (cpRNFLT) and major clinical markers of renal function.

**Additional file 2: Table S1.** Sectoral multivariable linear regression analyses for cardiometabolic biomarkers and cpRNFLT in all subjects with further adjustment for SD-OCT-derived clinical and subclinical ONH abnormalities (including glaucoma and other ONH diseases), as well as patient-reported glaucoma diagnosis and medication (N = 8952).

**Additional file 3: Table S2.** Sectoral analyses derived from cpRNFLT in all subjects with further adjustment for high sensitivity c-reactive protein (N = 8,952).

**Additional file 4: Table S3.** Baseline characteristics of the entire study population stratified by CKD risk status.

**Additional file 5: Table S4.** Baseline characteristics of the entire study population stratified by statin treatment.

**Additional file 6: Table S5.** Sectoral multivariable linear regression analyses for lipid markers and cpRNFLT in all subjects stratified by statin treatment.

**Additional file 7: Table S6.** Bayesian information criterion difference (ΔBIC) for the investigation of potential mediating effects of smoking status on the association between cpRNFLT and the lipid profile.

**Additional file 8: Table S7.** Sectoral analyses derived from cpRNFLT in patients with optic nerve head abnormalities, self-reported glaucoma diagnosis, and/or glaucoma medication (N = 1,180).

## Data Availability

Raw data cannot be shared publicly because of consent restrictions of LIFE-Adult participants. Data are available after an approved project agreement from the LIFE Leipzig Research Center for Civilization Diseases. Please contact Dr. Matthias Nüchter (Head of Managing Office, contact via matthias.nuechter[at]life.uni-leipzig.de) for data access requests.

## Abbreviations

Apo: Apolipoprotein
BIC: Bayesian information criterion
BMI: Body mass index
CKD: Chronic kidney disease
cpRNFLT: Circumpapillary RNFL thickness
eGFR: Estimated glomerular filtration rate
ESKD: End-stage kidney disease
FDR: False discovery rate
G: Global
HbA1c: Glycated hemoglobin A1c
HDL: High-density lipoprotein
hsCRP: High-sensitivity C-reactive protein
LDL: Low-density lipoprotein
Lp(a): Lipoprotein(a)
N: Nasal
NI: Infero-nasal
NS: Supero-nasal sector
RNFL: Retinal nerve fiber layer
SD-OCT: Spectral domain optical coherence tomography
T: Temporal sector
TG: Triglycerides
TI: Infero-temporal
TS: Supero-temporal sector
