# Supplementary material for: Renal function and lipid metabolism are major predictors of circumpapillary retinal nerve fiber layer thickness—the LIFE-Adult Study
Source: BMC Med. 2021 Sep 7;19:202. doi: 10.1186/s12916-021-02064-8 (PMC8422631; doi:10.1186/s12916-021-02064-8)
Supplement: Supplementary file 1 — Additional file 1: Figure S1. Scatterplots illustrating the relationship between global circumpapillary retinal nerve fiber layer thickness (cpRNFLT) and major clinical markers of renal function. [file 12916_2021_2064_MOESM1_ESM.docx]

**

**

**Supplementary Figure S1.**

Unadjusted scatterplots illustrating the relationship between global circumpapillary retinal nerve fiber layer thickness (cpRNFLT) and major clinical markers of renal function, i.e. **(A)** Cystatin C-based estimated glomerular filtration rate (eGFR_Cys_), and lipid profile, i.e. **(B)** High-density lipoprotein (HDL) cholesterol and **(C)** non-HDL cholesterol. A linear regression analysis for each of the three scatterplots was performed and the respective trendlines, as well as equation and the p values, are depicted.
