# Supplementary material for: Renal function and lipid metabolism are major predictors of circumpapillary retinal nerve fiber layer thickness—the LIFE-Adult Study
Source: BMC Med. 2021 Sep 7;19:202. doi: 10.1186/s12916-021-02064-8 (PMC8422631; doi:10.1186/s12916-021-02064-8)
Supplement: Supplementary file 2 — Additional file 2: Table S1. Sectoral multivariable linear regression analyses for cardiometabolic biomarkers and cpRNFLT in all subjects with further adjustment for SD-OCT-derived clinical and subclinical ONH abnormalities (including glaucoma and other ONH diseases), as well as patient-reported glaucoma diagnosis and medication (N = 8952). [file 12916_2021_2064_MOESM2_ESM.docx]

| **Supplementary Table S1:** Sectoral analyses derived from cpRNFLT in all subjects with further adjustment for SD-OCT-derived clinical and subclinical ONH abnormalities (including glaucoma and other ONH diseases), as well as patient-reported glaucoma diagnosis and medication (N = 8,952) | | | | | | | | | | | | | | |
| --- | --- | --- | --- | --- | --- | --- | --- | --- | --- | --- | --- | --- | --- | --- |
| **Sectors** | **Global** | | **T** | | **TS** | | **TI** | | **N** | | **NS** | | **NI** | |
|  | **B** | **p_adjusted_** | **B** | **p_adjusted_** | **B** | **p_adjusted_** | **B** | **p_adjusted_** | **B** | **p_adjusted_** | **B** | **p_adjusted_** | **B** | **p_adjusted_** |
| Diabetes | -0.69 | 0.122 | -0.75 | 0.147 | -0.95 | 0.232 | **-1.84** | **0.032** | -0.52 | 0.388 | -0.33 | 0.730 | 0.06 | 0.926 |
| Smoking status | **0.59** | **0.046** | **-0.74** | **0.046** | 0.41 | 0.441 | 0.65 | 0.271 | **1.05** | **0.031** | **1.31** | **0.046** | **1.71** | **0.022** |
| Hypertension | -0.39 | 0.273 | -0.26 | 0.671 | 0.02 | 0.970 | -0.80 | 0.273 | -0.82 | 0.157 | -0.13 | 0.970 | -0.10 | 0.970 |
| BMI (kg/m^2^) | **0.06** | **0.029** | -0.03 | 0.266 | 0.08 | 0.152 | 0.07 | 0.177 | 0.04 | 0.241 | **0.14** | **0.016** | **0.15** | **0.010** |
| WHR | 2.22 | 0.277 | -3.64 | 0.195 | 1.26 | 0.711 | 1.26 | 0.711 | 3.78 | 0.229 | 8.55 | 0.123 | 6.45 | 0.195 |
| SBP (mmHg) | -0.01 | 0.202 | -0.02 | 0.202 | -0.03 | 0.202 | -0.02 | 0.202 | -0.01 | 0.260 | 0.00 | 0.743 | 0.02 | 0.202 |
| DBP (mmHg) | 0.00 | 0.997 | -0.02 | 0.627 | 0.01 | 0.997 | 0.01 | 0.997 | 0.00 | 0.997 | -0.03 | 0.627 | 0.05 | 0.259 |
| Cystatin C (mg/l) | **-2.12** | **0.001** | **-2.01** | **0.007** | **-2.94** | **0.009** | **-4.36** | **0.001** | -0.74 | 0.363 | -1.14 | 0.363 | **-3.18** | **0.009** |
| eGFR_Cys_ (ml/min per 1.73m²) | **0.03** | **<0.001** | **0.03** | **0.001** | **0.04** | **0.009** | **0.06** | **<0.001** | 0.01 | 0.273 | 0.01 | 0.723 | **0.04** | **0.010** |
| Albumin-creatinine ratio (mg/g) | 0.00 | 0.905 | 0.00 | 0.905 | 0.00 | 0.905 | 0.00 | 0.905 | 0.00 | 0.793 | 0.00 | 0.905 | 0.00 | 0.905 |
| eGFR category (G1-G5) | **-0.91** | **<0.001** | **-1.02** | **<0.001** | **-1.32** | **0.001** | **-2.03** | **<0.001** | -0.17 | 0.564 | -0.31 | 0.546 | **-1.25** | **0.005** |
| Presence of CKD (yes/no) | **-1.97** | **<0.001** | **-1.10** | **0.034** | **-2.46** | **0.002** | **-5.10** | **<0.001** | -0.88 | 0.132 | -1.48 | 0.091 | **-2.71** | **0.002** |
| Fasting glucose (mmol/l) | 0.10 | 0.563 | -0.11 | 0.563 | 0.00 | 0.998 | -0.11 | 0.687 | 0.23 | 0.445 | 0.36 | 0.445 | 0.27 | 0.491 |
| Fasting insulin (pmol/l) | 0.00 | 0.167 | 0.00 | 0.345 | 0.01 | 0.102 | 0.00 | 0.226 | 0.00 | 0.948 | 0.01 | 0.100 | 0.00 | 0.948 |
| HbA1c (%) | 0.22 | 0.493 | 0.31 | 0.493 | 0.01 | 0.970 | -0.02 | 0.970 | 0.44 | 0.493 | -0.30 | 0.668 | 0.45 | 0.493 |
| Total cholesterol (mmol/l) | **0.40** | **0.001** | 0.02 | 0.888 | **0.60** | **0.006** | 0.34 | 0.124 | 0.28 | 0.097 | **0.72** | **0.003** | **0.99** | **<0.001** |
| HDL cholesterol (mmol/l) | -0.53 | 0.070 | -0.10 | 0.886 | **-1.19** | **0.048** | **-1.21** | **0.048** | 0.02 | 0.951 | **-1.24** | **0.048** | -0.37 | 0.684 |
| Non-HDL cholesterol (mmol/l) | **0.47** | **<0.001** | 0.04 | 0.743 | **0.77** | **<0.001** | **0.52** | **0.015** | 0.26 | 0.094 | **0.89** | **<0.001** | **1.00** | **<0.001** |
| LDL cholesterol (mmol/l) | **0.50** | **<0.001** | 0.22 | 0.119 | **0.71** | **0.003** | **0.69** | **0.005** | 0.27 | 0.119 | **0.55** | **0.033** | **1.08** | **<0.001** |
| TG (mmol/l) | 0.21 | 0.094 | -0.17 | 0.262 | 0.43 | 0.078 | 0.09 | 0.656 | 0.15 | 0.404 | **0.67** | **0.017** | 0.50 | 0.078 |
| ApoA1 (g/l) | -0.59 | 0.253 | -0.98 | 0.165 | -1.32 | 0.206 | -1.69 | 0.165 | -0.13 | 0.950 | 0.05 | 0.950 | 0.47 | 0.808 |
| ApoB (g/l) | **1.96** | **<0.001** | 0.11 | 0.832 | **2.95** | **<0.001** | **2.22** | **0.011** | **1.37** | **0.029** | **3.73** | **<0.001** | **3.94** | **<0.001** |
| Lp(a) (g/l) | 0.26 | 0.886 | 0.26 | 0.886 | -0.09 | 0.886 | 0.49 | 0.886 | 0.09 | 0.886 | 0.64 | 0.886 | 0.22 | 0.886 |
| hsCRP (mg/l) | 0.02 | 0.518 | 0.00 | 0.993 | 0.04 | 0.518 | -0.01 | 0.890 | 0.02 | 0.618 | 0.06 | 0.518 | 0.06 | 0.518 |
| IL-6 (ng/l) | 0.00 | 0.990 | -0.01 | 0.900 | 0.04 | 0.900 | -0.03 | 0.900 | -0.03 | 0.900 | 0.07 | 0.900 | 0.00 | 0.990 |
| ALAT (µkat/l) | 0.20 | 0.936 | -0.44 | 0.936 | -0.06 | 0.991 | 0.54 | 0.936 | 0.27 | 0.936 | -0.01 | 0.991 | 1.44 | 0.812 |
| ASAT (µkat/l) | -0.49 | 0.663 | -0.87 | 0.663 | -0.21 | 0.920 | -1.06 | 0.663 | -0.73 | 0.663 | 0.57 | 0.877 | 0.12 | 0.920 |
| AP (µkat/l) | -0.23 | 0.953 | -0.36 | 0.953 | -0.57 | 0.953 | -0.10 | 0.953 | -0.26 | 0.953 | -0.04 | 0.953 | 0.07 | 0.953 |
| GGT (µkat/l) | 0.00 | 0.995 | -0.31 | 0.252 | -0.19 | 0.684 | 0.04 | 0.995 | -0.14 | 0.684 | 0.31 | 0.671 | **0.79** | **0.049** |

**Supplementary Table S1.**

**Sectoral multivariable linear regression analyses for cardiometabolic biomarkers and cpRNFLT in all subjects with further adjustment for SD-OCT-derived clinical and subclinical ONH abnormalities (including glaucoma and other ONH diseases), as well as patient-reported glaucoma diagnosis and medication (N = 8,952).** For each of the six cpRNFL sectors, a linear regression model was calculated with age, sex, measurement radius, SD-OCT-derived clinical and subclinical ONH abnormalities (including glaucoma and other ONH diseases, patient-reported glaucoma diagnosis and medication), as well as the respective biomarker, as regressors. Unstandardized B coefficients, i.e. slope, and corresponding p values (corrected for multiple testing based on the false discovery rate method) for the respective cardiometabolic biomarkers are depicted. Abbreviations are indicated in Table 1 and 2. p values **marked in bold** indicate significant association in multivariate analysis.
