# Supplementary material for: Renal function and lipid metabolism are major predictors of circumpapillary retinal nerve fiber layer thickness—the LIFE-Adult Study
Source: BMC Med. 2021 Sep 7;19:202. doi: 10.1186/s12916-021-02064-8 (PMC8422631; doi:10.1186/s12916-021-02064-8)
Supplement: Supplementary file 3 — Additional file 3: Table S2. Sectoral analyses derived from cpRNFLT in all subjects with further adjustment for high sensitivity c-reactive protein (N = 8,952). [file 12916_2021_2064_MOESM3_ESM.docx]

| **Supplementary Table S2:** Sectoral analyses derived from cpRNFLT in all subjects with further adjustment for high sensitivity c-reactive protein (N = 8,952) | | | | | | | | | | | | | | |
| --- | --- | --- | --- | --- | --- | --- | --- | --- | --- | --- | --- | --- | --- | --- |
| **Sectors** | **Global** | | **T** | | **TS** | | **TI** | | **N** | | **NS** | | **NI** | |
|  | **B** | **p_adjusted_** | **B** | **p_adjusted_** | **B** | **p_adjusted_** | **B** | **p_adjusted_** | **B** | **p_adjusted_** | **B** | **p_adjusted_** | **B** | **p_adjusted_** |
| Diabetes | -0.79 | 0.066 | -0.79 | 0.123 | -1.17 | 0.123 | **-1.94** | **0.025** | -0.54 | 0.368 | -0.59 | 0.465 | -0.03 | 0.961 |
| Smoking status | 0.54 | 0.078 | -0.74 | 0.067 | 0.37 | 0.500 | 0.63 | 0.302 | **1.03** | **0.039** | 1.22 | 0.067 | **1.52** | **0.039** |
| Hypertension | -0.43 | 0.234 | -0.25 | 0.729 | 0.00 | 0.996 | -0.81 | 0.234 | -0.89 | 0.094 | -0.30 | 0.799 | -0.15 | 0.895 |
| BMI (kg/m^2^) | 0.05 | 0.061 | -0.03 | 0.331 | 0.07 | 0.192 | 0.08 | 0.187 | 0.04 | 0.331 | **0.12** | **0.046** | **0.14** | **0.030** |
| WHR | 1.61 | 0.511 | -3.81 | 0.262 | 0.06 | 0.986 | 0.87 | 0.937 | 3.37 | 0.326 | 7.71 | 0.252 | 5.16 | 0.326 |
| SBP (mmHg) | -0.01 | 0.258 | -0.02 | 0.235 | -0.03 | 0.235 | -0.02 | 0.258 | -0.01 | 0.269 | 0.00 | 0.784 | 0.02 | 0.235 |
| DBP (mmHg) | 0.00 | 0.926 | -0.02 | 0.571 | 0.00 | 0.926 | 0.00 | 0.926 | 0.00 | 0.926 | -0.03 | 0.571 | 0.05 | 0.362 |
| Cystatin C (mg/l) | **-2.32** | **<0.001** | **-2.09** | **0.004** | **-3.28** | **0.004** | **-4.52** | **<0.001** | -0.86 | 0.291 | -1.52 | 0.232 | **-3.52** | **0.004** |
| eGFR_Cys_ (ml/min per 1.73m²) | **0.03** | **<0.001** | **0.03** | **0.001** | **0.05** | **0.003** | **0.06** | **<0.001** | 0.02 | 0.187 | 0.01 | 0.458 | **0.05** | **0.004** |
| Albumin-creatinine ratio (mg/g) | 0.00 | 0.892 | 0.00 | 0.892 | 0.00 | 0.892 | 0.00 | 0.892 | 0.00 | 0.771 | 0.00 | 0.892 | 0.00 | 0.892 |
| eGFR category (G1-G5) | **-0.97** | **<0.001** | **-1.05** | **<0.001** | **-1.42** | **0.001** | **-2.08** | **<0.001** | -0.21 | 0.484 | -0.43 | 0.367 | **-1.35** | **0.003** |
| Presence of CKD (yes/no) | **-2.12** | **<0.001** | **-1.16** | **0.026** | **-2.81** | **0.001** | **-5.26** | **<0.001** | -0.94 | 0.110 | **-1.79** | **0.040** | **-2.92** | **0.001** |
| Fasting glucose (mmol/l) | 0.02 | 0.815 | -0.15 | 0.460 | -0.14 | 0.575 | -0.23 | 0.460 | 0.19 | 0.460 | 0.26 | 0.460 | 0.19 | 0.519 |
| Fasting insulin (pmol/l) | 0.00 | 0.416 | 0.00 | 0.458 | 0.00 | 0.280 | 0.00 | 0.416 | 0.00 | 0.818 | 0.01 | 0.273 | 0.00 | 0.818 |
| HbA1c (%) | 0.17 | 0.576 | 0.30 | 0.576 | -0.09 | 0.886 | -0.06 | 0.886 | 0.41 | 0.576 | -0.41 | 0.576 | 0.40 | 0.576 |
| Total cholesterol (mmol/l) | **0.43** | **<0.001** | 0.04 | 0.772 | **0.64** | **0.003** | 0.36 | 0.102 | 0.29 | 0.088 | **0.76** | **0.002** | **1.02** | **<0.001** |
| HDL cholesterol (mmol/l) | -0.55 | 0.063 | -0.14 | 0.778 | **-1.25** | **0.044** | **-1.34** | **0.044** | 0.03 | 0.944 | -1.23 | 0.054 | -0.33 | 0.765 |
| Non-HDL cholesterol (mmol/l) | **0.49** | **<0.001** | 0.06 | 0.613 | **0.82** | **<0.001** | **0.56** | **0.009** | 0.27 | 0.081 | **0.91** | **<0.001** | **1.01** | **<0.001** |
| LDL cholesterol (mmol/l) | **0.52** | **<0.001** | 0.24 | 0.100 | **0.77** | **0.002** | **0.71** | **0.004** | 0.28 | 0.100 | **0.59** | **0.022** | **1.09** | **<0.001** |
| TG (mmol/l) | 0.21 | 0.091 | -0.16 | 0.304 | 0.44 | 0.082 | 0.11 | 0.616 | 0.15 | 0.394 | **0.67** | **0.018** | 0.50 | 0.082 |
| ApoA1 (g/l) | -0.61 | 0.244 | -1.03 | 0.133 | -1.39 | 0.182 | -1.82 | 0.133 | -0.13 | 0.908 | 0.10 | 0.908 | 0.56 | 0.714 |
| ApoB (g/l) | **1.90** | **<0.001** | 0.14 | 0.791 | **2.91** | **0.001** | **2.15** | **0.016** | **1.29** | **0.042** | **3.62** | **<0.001** | **3.79** | **<0.001** |
| Lp(a) (g/l) | 0.19 | 0.913 | 0.22 | 0.913 | -0.25 | 0.913 | 0.40 | 0.913 | 0.06 | 0.913 | 0.54 | 0.913 | 0.16 | 0.913 |
| hsCRP (mg/l) | - | - | - | - | - | - | - | - | - | - | - | - | - | - |
| IL-6 (ng/l) | -0.01 | 0.728 | -0.02 | 0.728 | 0.03 | 0.728 | -0.03 | 0.728 | -0.04 | 0.728 | 0.05 | 0.728 | -0.02 | 0.728 |
| ALAT (µkat/l) | 0.15 | 0.916 | -0.41 | 0.916 | -0.09 | 0.916 | 0.53 | 0.916 | 0.19 | 0.916 | -0.12 | 0.916 | 1.31 | 0.916 |
| ASAT (µkat/l) | -0.49 | 0.679 | -0.82 | 0.679 | -0.23 | 0.925 | -1.02 | 0.679 | -0.77 | 0.679 | 0.52 | 0.920 | 0.11 | 0.925 |
| AP (µkat/l) | -0.20 | 0.962 | -0.31 | 0.962 | -0.51 | 0.962 | 0.13 | 0.965 | -0.28 | 0.962 | -0.08 | 0.965 | 0.03 | 0.965 |
| GGT (µkat/l) | 0.00 | 0.999 | -0.30 | 0.302 | -0.19 | 0.685 | 0.07 | 0.933 | -0.15 | 0.685 | 0.30 | 0.685 | 0.77 | 0.062 |

**Supplementary Table S2.**

**Sectoral analyses derived from cpRNFLT in all subjects with further adjustment for high sensitivity c-reactive protein (N = 8,952).** For each of the six cpRNFL sectors, a linear regression model was calculated with age, sex, and measurement radius, high sensitivity C-reactive protein, as well as the respective biomarker, as regressors. Unstandardized B coefficients, i.e. slope, and corresponding p values (corrected for multiple testing based on the false discovery rate method) for the respective cardiometabolic biomarkers are depicted. Abbreviations are indicated in Table 1 and 2. p values **marked in bold** indicate significant association in multivariate analysis.
