# Supplementary material for: Renal function and lipid metabolism are major predictors of circumpapillary retinal nerve fiber layer thickness—the LIFE-Adult Study
Source: BMC Med. 2021 Sep 7;19:202. doi: 10.1186/s12916-021-02064-8 (PMC8422631; doi:10.1186/s12916-021-02064-8)
Supplement: Supplementary file 4 — Additional file 4: Table S3. Baseline characteristics of the entire study population stratified by CKD risk status. [file 12916_2021_2064_MOESM4_ESM.docx]

| **Supplementary Table S3:** Baseline characteristics of the entire study population stratified by CKD risk status | | |
| --- | --- | --- |
|  | **Low**  **CKD risk** | **Moderate/High/Very high CKD risk** |
| Total N | 7,437 | 1,380 |
| Age (years) | 55.7 ± 11.9 | 67.0 ± 10.0* |
| Sex (female / male) N | 3,911 / 3,526 | 677 / 703* |
| Diabetes N (%) | 790 (10.6) | 426 (30.9)* |
| Smoker N (%) | 1,642 (22.1) | 207 (15.0)* |
| Hypertension N (%) | 3,303 (44.4) | 1,071 (77.6)* |
| Statin therapy N (%) | 746 (10.0) | 366 (26.5)* |
| Cystatin C (mg/l) | 0.9 ± 0.1 | 1.2 ± 0.4* |
| eGFR_Cys_ (ml/min per 1.73m²) | 90.5 ± 16.4 | 64.1 ± 20.8* |
| ACR (mg/g) | 6.6 ± 5.5 | 116.9 ± 422.1* |
| G (µm) | 94.9 ± 11.1 | 91.7 ± 12.4* |
| T (µm) | 71.1 ± 12.7 | 68.0 ± 13.7* |
| TS (µm) | 130.8 ± 20.3 | 125.1 ± 22.8* |
| TI (µm) | 140.2 ± 21.3 | 132.1 ± 23.3* |
| N (µm) | 70.4 ± 15.4 | 69.9 ± 17.1 |
| NS (µm) | 103.2 ± 22.3 | 100.3 ± 23.4* |
| NI (µm) | 102.3 ± 22.7 | 100.4 ± 24.7* |

**Supplementary Table S4.**

**Baseline characteristics of the entire study population stratified by CKD risk groups.** Abbreviations are indicated in Tables 1 and 2. Values for mean ± standard deviation are shown. p values were assessed by t test or Chi-squared test and corrected for multiple testing based on the false discovery rate method, respectively, and *indicates p<0.05 for low CKD risk vs. moderate/high/very high CKD risk.
