# Supplementary material for: Renal function and lipid metabolism are major predictors of circumpapillary retinal nerve fiber layer thickness—the LIFE-Adult Study
Source: BMC Med. 2021 Sep 7;19:202. doi: 10.1186/s12916-021-02064-8 (PMC8422631; doi:10.1186/s12916-021-02064-8)
Supplement: Supplementary file 5 — Additional file 5: Table S4. Baseline characteristics of the entire study population stratified by statin treatment. [file 12916_2021_2064_MOESM5_ESM.docx]

| **Supplementary Table S4:** Baseline characteristics of the entire study population stratified by statin treatment | | | |
| --- | --- | --- | --- |
|  | **Non-statin users** | **Statin users** | **p** |
| Total N | 7,822 | 1,130 | - |
| Age (years) | 56.1 ± 12.2 | 67.0 ± 10.0 | <0.001 |
| Sex (female / male) N | 4,242 / 3,580 | 423 / 707 | <0.001 |
| Diabetes N (%) | 816 (10.4) | 427 (37.8) | <0.001 |
| Smoker N (%) | 1,695 (21.7) | 180 (15.9) | <0.001 |
| Hypertension N (%) | 3,533 (45.2) | 911 (80.6) | <0.001 |
| Total cholesterol (mmol/l) | 5.7 ± 1.0 | 4.9 ± 1.0 | <0.001 |
| HDL cholesterol (mmol/l) | 1.6 ± 0.5 | 1.5 ± 0.4 | <0.001 |
| Non-HDL cholesterol (mmol/l) | 4.0 ± 1.1 | 3.5 ± 1.0 | <0.001 |
| LDL cholesterol (mmol/l) | 3.6 ± 0.9 | 2.9 ± 0.8 | <0.001 |
| TG (mmol/l) | 1.4 ± 1.0 | 1.7 ± 1.3 | <0.001 |
| ApoA1 (g/l) | 1.7 ± 0.3 | 1.6 ± 0.3 | <0.001 |
| ApoB (g/l) | 1.1 ± 0.3 | 1.0 ± 0.2 | <0.001 |
| Lp(a) (g/l) | 0.2 ± 0.3 | 0.3 ± 0.4 | <0.001 |
| G (µm) | 94.7 ± 11.2 | 92.4 ± 12.0 | <0.001 |
| T (µm) | 71.1 ± 12.8 | 67.7 ± 13.1 | <0.001 |
| TS (µm) | 130.5 ± 20.6 | 126.1 ± 22.3 | <0.001 |
| TI (µm) | 139.7 ± 21.3 | 133.9 ± 24.1 | <0.001 |
| N (µm) | 70.3 ± 15.5 | 70.4 ± 16.7 | 0.885 |
| NS (µm) | 102.9 ± 22.4 | 101.7 ± 23.3 | 0.123 |
| NI (µm) | 102.1 ± 22.9 | 101.5 ± 23.3 | 0.448 |

**Supplementary Table S5.**

**Baseline characteristics of the entire study population stratified by statin treatment.** Abbreviations are indicated in Tables 1 and 2. Values for mean ± standard deviation are shown. p values for statin users vs. non-statin users were assessed by t test or Chi-squared test and corrected for multiple testing based on the false discovery rate method, respectively.
