# Supplementary material for: Renal function and lipid metabolism are major predictors of circumpapillary retinal nerve fiber layer thickness—the LIFE-Adult Study
Source: BMC Med. 2021 Sep 7;19:202. doi: 10.1186/s12916-021-02064-8 (PMC8422631; doi:10.1186/s12916-021-02064-8)
Supplement: Supplementary file 6 — Additional file 6: Table S5. Sectoral multivariable linear regression analyses for lipid markers and cpRNFLT in all subjects stratified by statin treatment. [file 12916_2021_2064_MOESM6_ESM.docx]

| **Supplementary Table S5:** Sectoral multivariable linear regression analyses for lipid markers and cpRNFLT in all subjects stratified by statin treatment. | | | | | | | | | | | | | | | |
| --- | --- | --- | --- | --- | --- | --- | --- | --- | --- | --- | --- | --- | --- | --- | --- |
| **Sectors** | | **Global** | | **T** | | **TS** | | **TI** | | **N** | | **NS** | | **NI** | |
|  |  | **B** | **p_adjusted_** | **B** | **p_adjusted_** | **B** | **p_adjusted_** | **B** | **p_adjusted_** | **B** | **p_adjusted_** | **B** | **p_adjusted_** | **B** | **p_adjusted_** |
| **Statin users**  **(N = 1,130)** | Total cholesterol (mmol/l) | 0.80 | 0.090 | 0.18 | 0.665 | 1.14 | 0.170 | 0.74 | 0.371 | 0.60 | 0.327 | 1.87 | 0.061 | 1.16 | 0.170 |
|  | HDL cholesterol (mmol/l) | -0.21 | 0.919 | -1.87 | 0.369 | -0.64 | 0.919 | -1.12 | 0.919 | 1.55 | 0.674 | 0.81 | 0.919 | -0.17 | 0.919 |
|  | Non-HDL cholesterol (mmol/l) | 0.90 | **0.043** | 0.56 | 0.220 | 1.45 | 0.085 | 0.98 | 0.220 | 0.35 | 0.499 | 1.89 | **0.043** | 1.21 | 0.154 |
|  | LDL cholesterol (mmol/l) | 0.91 | 0.175 | 0.60 | 0.256 | 1.23 | 0.238 | 1.04 | 0.256 | 0.55 | 0.343 | 1.60 | 0.175 | 1.20 | 0.238 |
|  | TG (mmol/l) | 0.10 | 0.925 | 0.23 | 0.811 | 0.46 | 0.811 | 0.04 | 0.939 | -0.27 | 0.811 | 0.53 | 0.811 | -0.13 | 0.925 |
|  | ApoA1 (g/l) | 0.36 | 0.953 | -2.35 | 0.383 | 0.14 | 0.953 | -0.59 | 0.953 | 1.51 | 0.942 | 4.18 | 0.383 | 0.84 | 0.953 |
|  | ApoB (g/l) | 2.93 | 0.177 | 2.64 | 0.241 | 4.02 | 0.241 | 4.09 | 0.241 | 0.98 | 0.633 | 5.67 | 0.177 | 2.94 | 0.349 |
|  | Lp(a) (g/l) | -1.07 | 0.376 | 0.65 | 0.692 | -0.57 | 0.717 | -0.63 | 0.717 | -1.45 | 0.376 | -2.06 | 0.376 | -3.82 | 0.125 |
| **Non-statin users**  **(N = 7,822)** | Total cholesterol (mmol/l) | 0.32 | **0.017** | -0.13 | 0.431 | 0.45 | 0.088 | 0.11 | 0.634 | 0.29 | 0.134 | 0.67 | **0.017** | 1.05 | **<0.001** |
|  | HDL cholesterol (mmol/l) | -0.65 | **0.032** | -0.01 | 0.968 | -1.42 | **0.023** | -1.38 | **0.023** | -0.19 | 0.731 | -1.52 | **0.023** | -0.39 | 0.687 |
|  | Non-HDL cholesterol (mmol/l) | 0.41 | **0.001** | -0.11 | 0.433 | 0.66 | **0.004** | 0.35 | 0.141 | 0.29 | 0.103 | 0.88 | **0.001** | 1.02 | **<0.001** |
|  | LDL cholesterol (mmol/l) | 0.43 | **0.004** | 0.07 | 0.679 | 0.59 | **0.044** | 0.49 | 0.085 | 0.28 | 0.164 | 0.51 | 0.085 | 1.14 | **<0.001** |
|  | TG (mmol/l) | 0.25 | 0.095 | -0.22 | 0.151 | 0.44 | 0.095 | 0.12 | 0.626 | 0.26 | 0.151 | 0.70 | **0.039** | 0.64 | **0.039** |
|  | ApoA1 (g/l) | -0.81 | 0.144 | -0.90 | 0.149 | -1.70 | 0.138 | -2.04 | 0.109 | -0.37 | 0.644 | -0.56 | 0.644 | 0.39 | 0.666 |
|  | ApoB (g/l) | 1.63 | **0.001** | -0.57 | 0.311 | 2.38 | **0.013** | 1.28 | 0.185 | 1.48 | **0.037** | 3.58 | **0.001** | 4.00 | **<0.001** |
|  | Lp(a) (g/l) | 0.60 | 0.289 | 0.22 | 0.750 | 0.00 | 1.000 | 0.78 | 0.537 | 0.48 | 0.547 | 1.27 | 0.289 | 1.26 | 0.289 |

**Supplementary Table S6.**

**Sectoral multivariable linear regression analyses for lipid markers and cpRNFLT in all subjects stratified by statin treatment.** For each of the six cpRNFL sectors, a linear regression model was calculated with age, sex, and measurement radius, as well as the respective biomarker, as regressors. Unstandardized B coefficients and corresponding p values (corrected for multiple testing based on the false discovery rate method) for the respective cardiometabolic biomarkers are depicted. Abbreviations are indicated in Table 1 and 2. Unstandardized B coefficients and corresponding p values **marked in bold** indicate significant association in multivariate analysis.
