# Supplementary material for: Renal function and lipid metabolism are major predictors of circumpapillary retinal nerve fiber layer thickness—the LIFE-Adult Study
Source: BMC Med. 2021 Sep 7;19:202. doi: 10.1186/s12916-021-02064-8 (PMC8422631; doi:10.1186/s12916-021-02064-8)
Supplement: Supplementary file 7 — Additional file 7: Table S6. Bayesian information criterion difference (ΔBIC) for the investigation of potential mediating effects of smoking status on the association between cpRNFLT and the lipid profile. [file 12916_2021_2064_MOESM7_ESM.docx]

| **Supplementary Table S6:** Bayesian information criterion difference (ΔBIC) for the investigation of potential mediating effects of smoking status on the association between cpRNFLT and the lipid profile. | | | | | | | |
| --- | --- | --- | --- | --- | --- | --- | --- |
| **Sectors** | **Global** | **T** | **TS** | **TI** | **N** | **NS** | **NI** |
| Total cholesterol (mmol/l) | -5.6 | -4.2 | -8.6 | -8.0 | -2.6 | -4.6 | -2.6 |
| HDL cholesterol (mmol/l) | -6.3 | -3.8 | -8.9 | -8.4 | -2.6 | -5.4 | -2.8 |
| Non-HDL cholesterol (mmol/l) | -6.2 | -3.9 | -8.8 | -8.2 | -2.9 | -5.3 | -3.4 |
| LDL cholesterol (mmol/l) | -5.6 | -4.0 | -8.6 | -8.1 | -2.6 | -4.5 | -2.7 |
| TG (mmol/l) | -5.8 | -4.4 | -8.7 | -8.0 | -2.8 | -5.2 | -3.0 |
| ApoA1 (g/l) | -5.8 | -3.4 | -8.7 | -8.2 | -2.5 | -4.5 | -2.0 |
| ApoB (g/l) | -6.1 | -4.0 | -8.8 | -8.1 | -2.9 | -5.4 | -3.1 |
| Lp(a) (g/l) | -5.2 | -4.0 | -8.5 | -7.9 | -2.3 | -4.4 | -1.8 |

**Supplementary Table S7**

**Bayesian information criterion difference (**Δ**BIC) for the investigation of potential mediating effects of smoking status on the association between cpRNFLT and the lipid profile.** For each of the six cpRNFL sectors, two different linear regression models were calculated with age, sex, measurement radius, and the respective lipid marker as regressors (model A), as well as an additional model comprising of model A + smoking status (model B). The BIC difference (ΔBIC) was calculated by ΔBIC = BIC_model A_ - BIC_model B_. ΔBIC values are depicted and a ΔBIC > 2 was regarded as statistically relevant. Abbreviations are indicated in Table 1 and 2.
