# Supplementary material for: Renal function and lipid metabolism are major predictors of circumpapillary retinal nerve fiber layer thickness—the LIFE-Adult Study
Source: BMC Med. 2021 Sep 7;19:202. doi: 10.1186/s12916-021-02064-8 (PMC8422631; doi:10.1186/s12916-021-02064-8)
Supplement: Supplementary file 8 — Additional file 8: Table S7. Sectoral analyses derived from cpRNFLT in patients with optic nerve head abnormalities, self-reported glaucoma diagnosis, and/or glaucoma medication (N = 1,180). [file 12916_2021_2064_MOESM8_ESM.docx]

| **Supplementary Table S7:** Sectoral analyses derived from cpRNFLT in patients with optic nerve head abnormalities, self-reported glaucoma diagnosis, and/or glaucoma medication (N = 1,180) | | | | | | | | | | | | | | | |
| --- | --- | --- | --- | --- | --- | --- | --- | --- | --- | --- | --- | --- | --- | --- | --- |
| **Sectors** | | **Global** | | **T** | | **TS** | | **TI** | | **N** | | **NS** | | **NI** | |
|  |  | **B** | **p_adjusted_** | **B** | **p_adjusted_** | **B** | **p_adjusted_** | **B** | **p_adjusted_** | **B** | **p_adjusted_** | **B** | **p_adjusted_** | **B** | **p_adjusted_** |
| **Patients with glaucoma**  **(N = 1,180)** | Total cholesterol (mmol/l) | **1.54** | **0.002** | 0.59 | 0.188 | **1.99** | **0.022** | **2.35** | **0.008** | **1.20** | **0.030** | **2.74** | **0.002** | **1.60** | **0.030** |
|  | HDL cholesterol (mmol/l) | -0.96 | 0.462 | -1.27 | 0.446 | -4.36 | 0.109 | -2.31 | 0.446 | 1.35 | 0.446 | -1.12 | 0.588 | -0.11 | 0.947 |
|  | Non-HDL cholesterol (mmol/l) | **1.63** | **<0.001** | 0.81 | 0.069 | **2.68** | **0.001** | **2.66** | **0.001** | 0.89 | 0.079 | **2.78** | **<0.001** | **1.54** | **0.036** |
|  | LDL cholesterol (mmol/l) | **1.74** | **0.002** | **1.08** | **0.036** | **2.45** | **0.008** | **3.05** | **0.002** | 1.01 | 0.076 | **2.54** | **0.004** | **1.68** | **0.036** |
|  | TG (mmol/l) | 0.67 | 0.368 | 0.03 | 0.953 | 1.85 | 0.243 | 0.95 | 0.394 | 0.25 | 0.775 | 1.03 | 0.368 | 0.99 | 0.368 |
|  | ApoA1 (g/l) | -1.14 | 0.638 | -3.25 | 0.149 | -5.98 | 0.149 | -3.78 | 0.333 | 2.71 | 0.333 | 0.20 | 0.941 | 1.55 | 0.638 |
|  | ApoB (g/l) | **6.50** | **<0.001** | 3.84 | 0.038 | **9.80** | **0.003** | **10.96** | **0.001** | 3.24 | 0.114 | **11.12** | **<0.001** | **5.88** | **0.042** |
|  | Lp(a) (g/l) | 0.54 | 0.797 | 0.97 | 0.797 | 2.95 | 0.632 | 2.69 | 0.632 | -0.65 | 0.797 | 0.58 | 0.797 | -2.63 | 0.632 |

**Supplementary Table S3.**

**Sectoral analyses derived from cpRNFLT in patients with** **optic nerve head abnormalities, self-reported glaucoma diagnosis, and/or glaucoma medication (N = 1,180).** For each of the six cpRNFL sectors, a linear regression model was calculated with age, sex, and measurement radius, as well as the respective lipid marker, as regressors. Unstandardized B coefficients and corresponding p values (corrected for multiple testing based on the false discovery rate method) for the respective lipid markers are depicted. Abbreviations are indicated in Table 1 and 2. Unstandardized B coefficients and corresponding p values **marked in bold** indicate significant association in multivariate analysis.
